# High Salt Intake Augments Excitability of PVN Neurons in Rats: Role of the Endoplasmic Reticulum Ca^2+^ Store

**DOI:** 10.3389/fnins.2017.00182

**Published:** 2017-04-06

**Authors:** Robert A. Larson, Andrew D. Chapp, Le Gui, Michael J. Huber, Zixi Jack Cheng, Zhiying Shan, Qing-Hui Chen

**Affiliations:** ^1^Department of Kinesiology and Integrative Physiology, Michigan Technological UniversityHoughton, MI, USA; ^2^Department of Cardiology, Affiliated Hospital of Nantong UniversityNantong, China; ^3^Biomolecular Science Center, Burnett School of Biomedical Sciences, College of Medicine, University of Central FloridaOrlando, FL, USA

**Keywords:** high salt diet, hypertension, paraventricular nucleus, sympathetic nerve activity, endoplasmic reticulum

## Abstract

High salt (HS) intake sensitizes central autonomic circuitry leading to sympathoexcitation. However, its underlying mechanisms are not fully understood. We hypothesized that inhibition of PVN endoplasmic reticulum (ER) Ca^2+^ store function would augment PVN neuronal excitability and sympathetic nerve activity (SNA). We further hypothesized that a 2% (NaCl) HS diet for 5 weeks would reduce ER Ca^2+^ store function and increase excitability of PVN neurons with axon projections to the rostral ventrolateral medulla (PVN-RVLM) identified by retrograde label. PVN microinjection of the ER Ca^2+^ ATPase inhibitor thapsigargin (TG) increased SNA and mean arterial pressure (MAP) in a dose-dependent manner in rats with a normal salt (NS) diet (0.4%NaCl). In contrast, sympathoexcitatory responses to PVN TG were significantly (*p* < 0.05) blunted in HS treated rats compared to NS treatment. In whole cell current-clamp recordings from PVN-RVLM neurons, graded current injections evoked graded increases in spike frequency. Maximum discharge was significantly augmented (*p* < 0.05) by HS diet compared to NS group. Bath application of TG (0.5 μM) increased excitability of PVN-RVLM neurons in NS (*p* < 0.05), yet had no significant effect in HS rats. Our data indicate that HS intake augments excitability of PVN-RVLM neurons. Inhibition of the ER Ca^2+^-ATPase and depletion of Ca^2+^ store likely plays a role in increasing PVN neuronal excitability, which may underlie the mechanisms of sympathoexcitation in rats with chronic HS intake.

## Introduction

Elevated dietary salt intake is a major contributor to the pathogenesis of cardiovascular disease (Appel et al., 2011; Kotchen et al., 2013; Oh et al., 2016). Interestingly, accumulating evidence indicates that the adverse effects of excess dietary salt on cardiovascular function may be independent of changes in arterial blood pressure (ABP) (Frohlich, 2007; Stocker et al., 2010; Appel et al., 2011; Kotchen et al., 2013; Cook et al., 2014). The central nervous system is an important mediator of cardiovascular disease including salt-sensitive hypertension through augmented sympathetic nerve activity (SNA) (King et al., 2007; Osborn et al., 2007; Elijovich et al., 2016). Evidence indicates that high salt intake sensitizes central autonomic circuits contributing to exaggerated SNA and ABP responses to stimuli in normotensive rats (Pawloski-Dahm and Gordon, 1993; Ito et al., 1999; Adams et al., 2007, 2008, 2009; Simmonds et al., 2014). Therefore, HS diet may create a predisposition whereby exaggerated sympathoexcitatory responses to physiological or social stressors contributes to the development of cardiovascular disease.

The hypothalamic paraventricular nucleus (PVN) is a prominent regulatory center for the sympathetic nervous system. Pre-sympathetic neurons in the PVN receive excitatory synaptic input from the forebrain circumventricular organs (CVO) (Miselis, 1981; Shi et al., 2008), and project to the excitatory centers in the brain stem and spinal cord that drive SNA (Chen and Toney, 2003). Studies have demonstrated that enhanced PVN neuronal activity supports the augmented SNA in several models of neurogenic hypertension (HTN) (Herzig et al., 1991; Allen, 2002; Bardgett et al., 2014); however, little is known regarding the effects of dietary salt on PVN neuronal excitability in normotensive animals.

Augmented PVN neuronal activity has been demonstrated in several models of salt-sensitive HTN through synaptic mechanisms including loss of GABA inhibition (Martin and Haywood, 1998), and augmented excitatory neurotransmitter signaling including glutamate and angiotensin II (Gabor and Leenen, 2012). Furthermore, alterations of intrinsic membrane properties that influence PVN neuronal excitability also play an important role in neurogenic HTN. Our lab (Larson et al., 2015a), and others (Pachuau et al., 2014) have recently demonstrated that dysfunction of small conductance Ca^2+^-activated K^+^ (SK) channels contribute to neurogenic HTN. Interestingly, we further demonstrated SK channel dysfunction in rats fed a 2% high salt (HS) diet even in the absence of hypertension.

Recent reports indicate that brain endoplasmic reticulum (ER) stress is a mediator of neurogenic HTN, yet the mechanisms remain unclear (Young et al., 2012, 2015; Chao et al., 2013). The ER serves as a Ca^2+^ store, and Ca^2+^ release from the ER is a prominent activator of SK channels to mediate neuronal excitability (Sah, 1996). We tested the hypothesis that inhibition of the ER Ca^2+^-ATPase and depletion of Ca^2+^ store from the ER with thapsigargin (TG) would augment SNA *in vivo* and neuronal excitability *in vitro*. Additionally, we further examined whether HS intake would augment excitability of PVN neurons and the inhibition of the ER Ca^2+^ uptake by TG on HS induced neuronal excitability and sympathoexcitation. Portions of this data have been presented in abstract form (Larson et al., 2014, 2015b).

## Methods

### Animals

Male Sprague-Dawley rats (*n* = 61, 250–350 g) purchased from Charles River Labs (Wilmington, MA) were housed in a temperature-controlled room (22–23C) with a 12:12 h light-dark cycle. Tap water was available *ad libitum*. Animals in the normal salt (NS) treatment group received standard laboratory chow (0.4% NaCl) *ad libitum* for 5 weeks whereas the HS treatment group was fed a 2% NaCl diet *ad libitum* for 5 weeks. All experimental and surgical procedures were carried out under the guidelines of the National Institutes of Health *Guide for the Care and Use of Laboratory Animals* with the approval of the Institutional Animal Care and Use Committee of Michigan Technological University.

### Microinjection experiment preparation

On the day of the experiment, rats were anesthetized with a mixture containing α–chlorolose (80 mg kg^−1^) and urethane (800 mg kg^−1^). Body temperature was maintained at 37°C with a water circulating pad. Catheters were implanted into the left femoral artery and vein in order to record arterial blood pressure (ABP) and administer drugs, respectively. Heart rate (HR) was obtained from the R-wave of the electrocardiogram (lead I). Rats were paralyzed with gallamine triethiodide, ventilated with O_2_-rich room air, and end-tidal PCO_2_ was monitored and maintained within normal limits (33–40 mmHg). Adequate depth of anesthesia was determined by lack of withdrawal reflex to noxious foot pinch prior to paralysis, and absence of pressor response to noxious foot pinch following paralysis. Supplemental doses of anesthesia equal to 10% of the initial dose were given as needed. All animals were allowed to stabilize at least 2 h following surgery.

### Recording of sympathetic nerve activity (SNA)

Rats were prepared for recording of splanchnic (SSNA) and renal (RSNA) SNA according to protocols previously described (Gui et al., 2012; Larson et al., 2015a). Briefly, a left flank incision was made and a left renal nerve and a postganglionic splanchnic nerve were separated from surrounding tissue. Nerve bundles were mounted on separate silver wire electrodes (0.127-mm diameter, A-M Systems) and covered with silicon-based impression material (Kwik-sil, WPI). The signal was directed to an alternating current amplifier (P511, Grass Technologies) equipped with half-amplitude filters (band pass, 100–1,000 Hz) and a 60-Hz notch filter. The processed signal was rectified, integrated (10-ms time constant), and digitized at a frequency of 5,000 Hz using a 1401 Micro3 analog-to-digital converter and Spike 2 software (version 7.04, Cambridge Electronic Design, Cambridge, UK).

### Hypothalamic paraventricular nucleus (PVN) microinjection

PVN microinjections were performed as previously described (Gui et al., 2012; Larson et al., 2015a). Animals were placed in a stereotaxic head frame and the skull was leveled between bregma and lambda. A small section of the skull was removed in order to expose the dura, and a single-barreled glass microinjector pipette was lowered vertically into the PVN. The stereotaxic coordinates used were as follows: 1.2–1.6 mm caudal to bregma, 0.5 mm lateral to midline, and 7.0–7.2 mm ventral to dura. After a 20-min baseline period, thapsigargin (TG; 100 nl), an inhibitor of the endoplasmic reticulum (ER) Ca^2+^ ATPase (Tocris), or vehicle (DMSO; 100 nl) was injected bilaterally into the PVN. The micropipette was withdrawn between injections and bilateral injections were separated by 2 min. Variables were recorded for 2 h following microinjection. At the conclusion of each experiment, Chicago blue dye (2% in saline, 100 nl) was injected into the PVN to mark the injection sites. Rats were decapitated and the brain was removed, postfixed in 4% paraformaldehyde, and then transferred to 30% sucrose-PBS. The hypothalamus, including the PVN area, was sliced in coronal sections, and microinjection sites were visualized under bright-field microscopy.

### Retrograde labeling

Five to seven days prior to neuronal excitability recording, PVN neurons were retrogradely labeled from the ipsilateral rostral ventrolateral medulla (RVLM) as previously described (Chen and Toney, 2009; Chen et al., 2010). Briefly, rats were anesthetized with isoflurane (3% in O_2_) and placed in a stereotaxic frame. The cerebellum was exposed through a small burr hole and a glass micropipette was lowered into the pressor region of the RVLM (coordinates: −12.7 mm caudal to bregma, 1.8 mm lateral to midline and 8.9 mm below the skull). A pneumatic pump was utilized to inject Flurospheres (150 nl, Life Technologies) into the RVLM. Animals received daily subcutaneous injection of penicillin G (30,000 units) and meloxicam (1 mg kg^−1^) for 3 days post-surgery. Tracer location was verified histologically post-mortem.

### Electrophysiology

Five to seven days post-retrograde tracer injection, rats were anesthetized with isoflurane (3% in O_2_) and decapitated. The brain was removed and chilled in ice-cold cutting solution containing (in mM): 206 sucrose, 2 KCl, 2 MgSO_4_, 1.25 NaH_2_PO_4_, 26 NaHCO_3_, 1 CaCl_2_, 1 MgCl_2_, 10 d-glucose, and 0.4 ascorbic acid, osmolarity 295–302 mosmol L^−1^ measured with an osmometer (Wescor), pH 7.3–7.4, continuously gassed with 95:5 CO_2_:O_2_ to maintain pH and pO_2_. A brain block was cut including the PVN region and affixed to a vibrating microtome (Leica VT 1000S; Leica, Nussloch, Germany). Coronal sections of 250 μm thickness were cut, and the slices transferred to a holding container of artificial cerebral spinal fluid (ACSF) maintained at 30°C, continuously gassed with 95:5 CO_2_:O_2_, containing (in mM): 125 NaCl, 2 KCl, 2 MgSO_4_, 1.25 NaH_2_PO_4_, 26 NaHCO_3_, 2 CaCl_2_, 10 d-glucose, and 0.4 ascorbic acid (osmolality: 295–302 mosmol L^−1^; pH 7.3–7.4) and allowed to recover for 1 h. Following recovery, slices were transferred to a glass-bottomed recording chamber and viewed through an upright microscope (E600FN, Nikon) equipped with DIC optics, epi-fluorescence, an infrared (IR) filter and an IR-sensitive video camera (C2400, Hamamatsu, Bridgewater, NJ) (Figure 6A). Patch electrodes were pulled from borosilicate glass capillaries and polished to a tip resistance of 4–8 MΩ. Electrodes were filled with a solution containing (in mM) 135 K-gluconate, 10 HEPES, 0.1 EGTA, 1.0 MgCl_2_, 1.0 NaCl, 2.0 Na_2_ATP, and 0.5 Na_2_GTP (osmolality: 280–285 mosmol L^−1^; pH 7.3). Upon achievement of a gigaohm seal and whole cell configuration, cell capacitance, access resistance and resting membrane potential (*V*_*m*_) were monitored to ensure stability. Cells that met the following criteria were included in data set: Action potential amplitude ≥ 50 mV from threshold to the peak, input resistance (*R*_input_) larger than 0.5 GΩ when hyperpolarizing current injections of −20 pA were delivered from a holding potential of −80 mV, resting *V*_*m*_ negative to −50 mV, and less than 20% change in series resistance during recording.

### Testing neuronal excitability

Neuronal excitability from NS and HS rats was studied in current-clamp mode. *V*_*m*_ was adjusted to −80 mV by injecting continuous negative current, and a series of square-wave current injections was delivered in increments of +25 pA, for a duration of 800 ms each. To determine action potential voltage threshold (*V*_*t*_) and depolarizing *R*_input_ below *V*_*t*_, ramp current injections (0.2 pA ms^−1^, 1,000 ms) were made from a potential of −80 mV. Effects of ER Ca^2+^ uptake inhibition with TG (0.5 μM) on neuronal excitability were determined by comparing current evoked *V*_*m*_ and spike frequency responses under control conditions, and following bath application of TG. Brain slices in the recording chamber were allowed at least 30 min of exposure time to the TG prior to recording. Slices were continuously perfused with the TG ACSF at ~2 mL per minute and gassed with 95:5 CO_2_:O_2_ and heated with an inline heating pen at 30°C. Recordings were made from different slices in the absence and presence of TG, and responses were recorded from separate groups of NS and HS PVN-RVLM neurons.

### Chemicals

All chemicals were obtained from Sigma-Aldrich (St Louis, MO) except for TTX (Tocris) and TG (Tocris).

### Data analysis

Summary data are expressed as mean ± SE. SSNA and RSNA were determined as an average of the rectified, integrated signal. Baseline values were obtained by averaging a 10-min window of data immediately prior to PVN microinjection. Responses to PVN microinjection were obtained by averaging a 2-min segment centered on the maximal response quantified after subtraction of background noise obtained following bolus injection of the ganglionic blocker hexamethonium (30 mg kg^−1^, IV). Dose dependent responses to PVN microinjection of TG, and the effect of TG on neuronal excitability and SFA in brain slice preparations from NS and HS rats was determined by one-way ANOVA with *post hoc* analysis determined by Newman-Keuls multiple comparison test. Sympathoexcitatory responses to PVN microinjection of TG in NS and HS rats were determined with unpaired *t*-tests. Difference were considered statistically significant at a critical value of *P* < 0.05.

## Results

### Depletion of PVN ER Ca^2+^ stores by TG augments SNA and ABP

**Figure 1:**
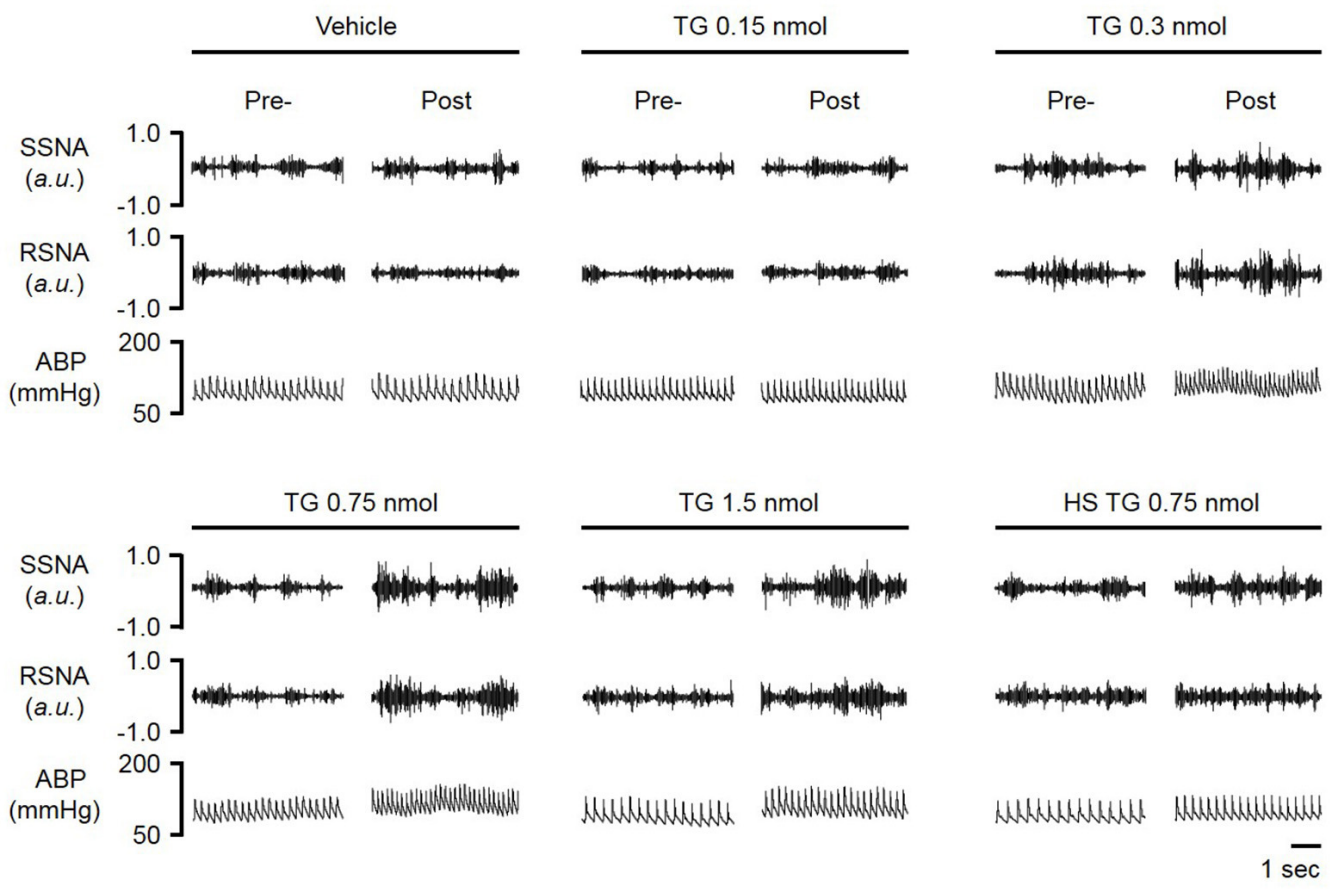
**Representative raw tracings of splanchnic sympathetic nerve activity (SSNA), renal sympathetic nerve activity (RSNA) and arterial blood pressure (ABP) in response to paraventricular nucleus (PVN) microinjection of graded doses (vehicle, 0.15, 0.30, 0.75, 1.5 nmol) of the ER Ca^2+^ ATPase inhibitor thapsigargin (TG); HS-high salt diet (2%)**.

**Figure 2:**
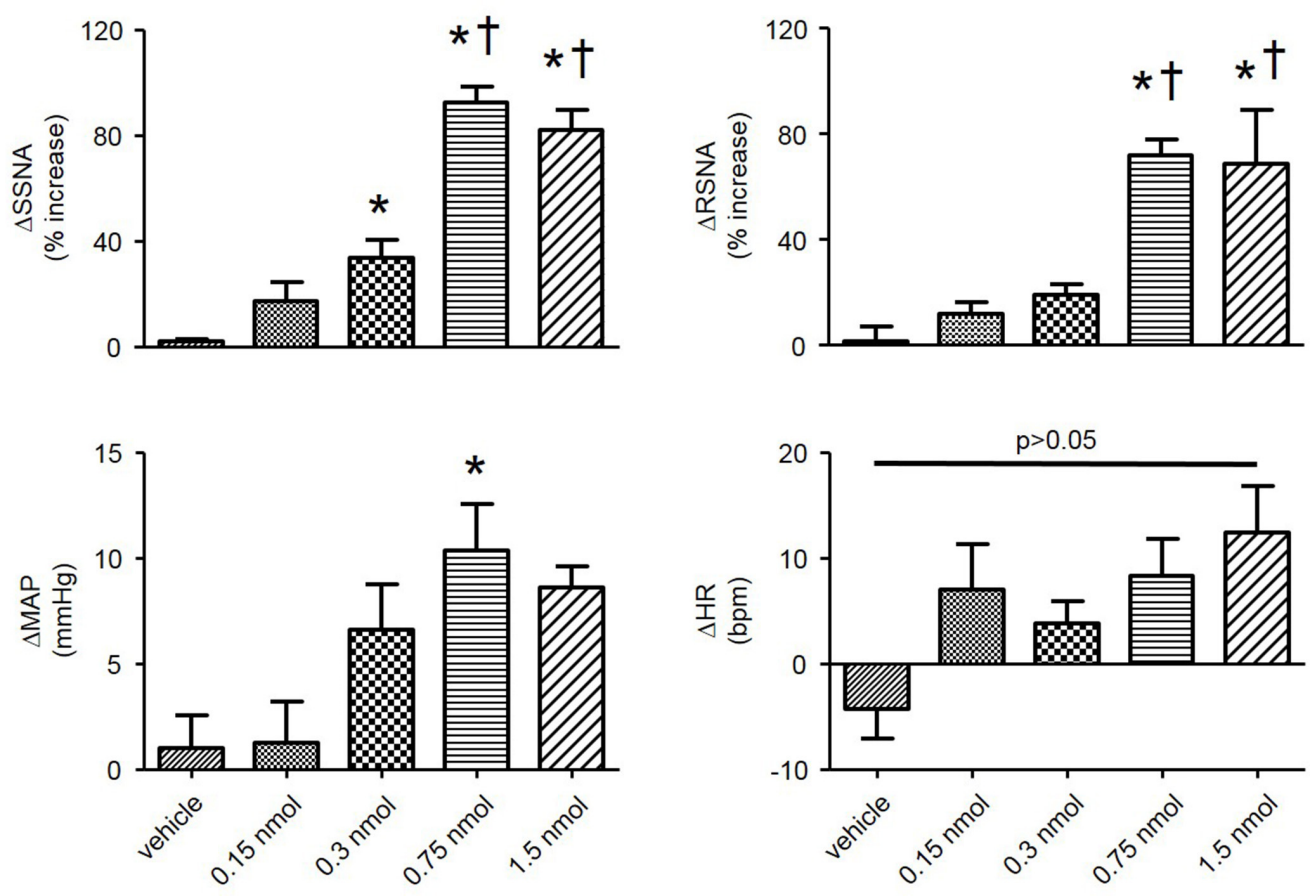
**Summary data showing changes in SSNA, RSNA, mean arterial pressure (MAP), and heart rate (HR) in response to bilateral microinjections of varying doses of TG (vehicle, *n* = 3; 0.15 nmol, *n* = 4; 0.3 nmol, *n* = 5; 0.75 nmol, *n* = 6; 1.5 nmol, *n* = 5) into the PVN**. ^*^P < 0.05 vs. vehicle; ^†^*P* < 0.05 vs. 0.3 nmol (1-way ANOVA Newman-Keuls multiple-comparison test).

In order to determine the contribution of the PVN neuronal ER Ca^2+^ stores in regulating SNA and ABP, we performed bilateral microinjection of TG into the PVN. PVN microinjection of TG elicited robust increases in SNA and ABP demonstrated in the raw trace data in Figure 1. Microinjection of TG (0.15 nmol, *n* = 4; 0.3 nmol, *n* = 5; 0.75 nmol, *n* = 6) increased splanchnic SNA (SSNA), renal SNA (RSNA), and MAP in a dose dependent manner. Maximum increases in SSNA, RSNA and mean arterial pressure (MAP) elicited by PVN TG (0.75 nmol/100 nl; *n* = 6) were 92 ± 6% (*P* < 0.05 vs. vehicle), 72 ± 6% (*P* < 0.05 vs. vehicle), and 10 ± 2 mmHg (*P* < 0.05 vs. vehicle), respectively as shown in Figure 2. Bilateral microinjection of vehicle (DMSO, 100 nL) into the PVN failed to elicit any significant response (*P* > 0.05 vs. baseline) in SNA, MAP or HR (Figures 1, 2). There were no significant differences in HR between any TG treatment dose and vehicle control (Figure 2). Bilateral microinjection of TG (0.75 nmol, 100 nl) outside of the PVN (~2.5 mm lateral to midline) failed to produce any significant change in SSNA (10.5 ± 8.1%), RSNA (4.4 ± 7.1%), or MAP (1 ± 1 mmHg) respectively, indicating that the effects of TG appear to be site specific (Table 1). In addition, the maximally effective dose of TG was injected into the femoral vein in order to exclude the possible influence of peripheral actions. IV administration of TG had no significant effect on resting SSNA, RSNA, MAP, and HR (Table 1).

**Table 1:** **Effect of injected compounds on resting MAP, HR, SSNA, and RSNA**.

**Injected compound**	***n***	**MAP, mmHg**		**HR, beats/min**		**SSNA**, μ**V**		**RSNA**, μ**V**	
		**Pre**	**Post**	**Pre**	**Post**	**Pre**	**Post**	**Pre**	**Post**
NS-TG	6	107 ± 6	117 ± 6^*^	350 ± 7	359 ± 6	0.03 ± 0.005	0.05 ± 0.008^*^	0.03 ± 0.009	0.05 ± 0.013^*^
0.75 nmol, PVN									
HS-TG	6	106 ± 4	114 ± 5^*^	325 ± 14	330 ± 15	0.04 ± 0.009	0.05 ± 0.010^*^	0.03 ± 0.006	0.03 ± 0.007^*^
0.75 nmol, PVN									
DMSO, PVN	3	111 ± 7	112 ± 6	352 ± 35	348 ± 34	0.03 ± 0.001	0.03 ± 0.005	0.02 ± 0.003	0.02 ± 0.004
Anatomy control (TG outside PVN)	5	111 ± 6	112 ± 6	337 ± 19	334 ± 22	0.02 ± 0.002	0.02 ± 0.002	0.04 ± 0.008	0.02 ± 0.007
TG intravenous	4	111 ± 8	107 ± 10	343 ± 23	344 ± 21	0.03 ± 0.003	0.02 ± 0.003	0.04 ± 0.009	0.02 ± 0.01

*Values are mean ± SE; n, number of rats; SSNA, splanchnic sympathetic nerve activity; RSNA, renal sympathetic nerve activity; MAP, mean arterial pressure; HR, heart rate; TG, thapsigargin; PVN, paraventricular nucleus*.

* *P < 0.05 vs. pre-treatment*.

### Sympathoexcitatory responses to PVN TG are attenuated by HS diet

**Figure 3:**
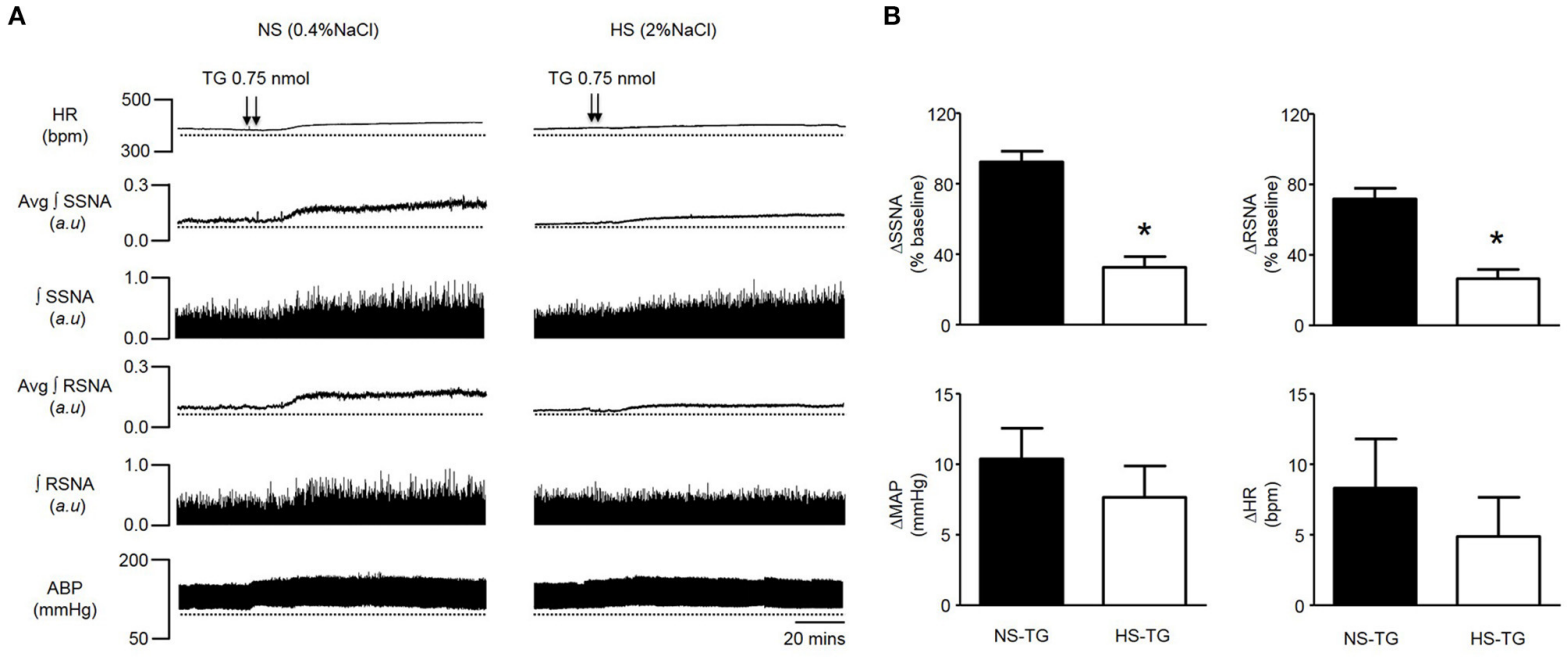
**(A)** Representative traces showing HR, SSNA, RSNA, and ABP responses to bilateral PVN microinjection of TG (0.75 nmol) in a rat on a 0.4% normal salt (NS) diet (left), and a 2% high salt (HS) (right). Bilateral PVN microinjection (100 nl each) of TG (arrowheads) markedly increased HR, SSNA, RSNA and ABP in a NS diet rat, whereas responses were attenuated in a rat fed a HS diet. **(B)** Summary data showing peak changes in SSNA, RSNA, MAP, and HR after bilateral PVN microinjection of TG (0.75 nmol) in normal salt (NS, *n* = 6) and high salt (HS, *n* = 6) rats. Note that SSNA and RSNA responses to PVN TG were significantly attenuated in HS rats compared to NS. ^*^P < 0.05 HS vs. NS (unpaired student *t*-test). Avg, average; ∫, integrated.

HS diet is known to increase the excitability of brainstem autonomic circuitry (Adams et al., 2007, 2009), but little is known regarding the effects of HS diet on PVN neuronal excitability. Therefore, we sought to determine whether HS diet influences sympathoexcitatory responses to PVN microinjection of TG. There was no significant difference in baseline MAP between NS and HS treatment groups (Table 1). Figure 3A, left, demonstrates representative raw tracings before, and after bilateral microinjection of TG from the NS treatment group. Figure 3A, right, demonstrates a representative response to bilateral PVN microinjection of TG (0.75 nmol, 100 nl) from the HS treatment group with a significantly attenuated response compared to NS. Microinjection of 0.75 nmol TG was utilized for comparison, as it was the minimum dose to elicit a maximum response in control rats (Figures 1, 2). Maximum increases in SSNA (33 ± 6%; *P* < 0.0001) and RSNA (26 ± 5%; *P* = 0.0002), were significantly attenuated compared to NS as demonstrated by summary data in Figure 3A. The average latency from microinjection of TG to the maximum response was not significantly different (*P* = 0.336) between NS (40 ± 5 min) and HS (35 ± 3 min) treatment groups. There were no significant differences in MAP (7 ± 2 mmHg; *P* = 0.208) and HR (5 ± 3 bpm; *p* = 0.223) between NS and HS treatment groups (Figure 3B).

### HS diet augments excitability of PVN-RVLM neurons

**Figure 4:**
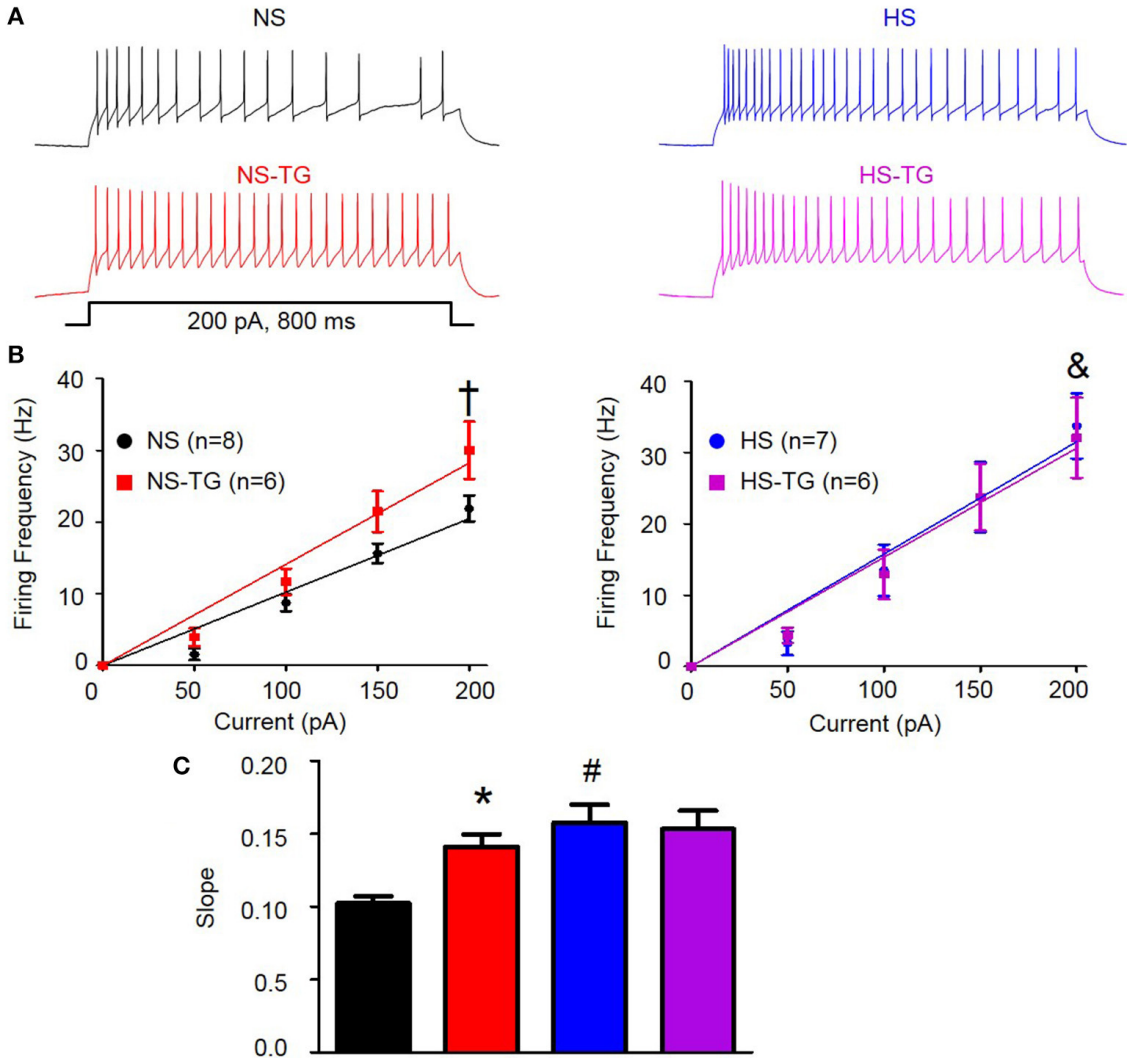
**Effect of ER Ca^**2+**^ uptake inhibition with TG on excitability of PVN-RVLM neurons from NS and HS rats. (A)** Voltage traces illustrating neuronal excitability in response to a 200 pA current injection in PVN-RVLM neurons from NS (left) and HS (right) rats in the absence (top, control) and presence (bottom left) of the ER Ca^2+^ ATPase inhibitor TG. **(B)** Linear response demonstrating the slope of firing frequency in response to graded current injection (0–200 pA) in PVN-RVLM neurons in the absence and presence of TG in NS (left) and HS (right) neurons. TG increased firing frequency in the NS group, but not HS. Note that firing frequency in response to 200 pA depolarizing current injection was increased in HS (right) compared to NS (left). **(C)** Summary data showing slope of firing frequency in response to graded current injection before and after bath application of TG in NS and HS rats. Inhibition of the ER Ca^2+^ store with TG augmented the slope in NS, but not HS neurons. Note that in control conditions, the slope was significantly greater in HS neurons. NS-normal salt; HS-high salt; TG-thapsigargin. ^†^*P* < 0.05 NS vs. NS –TG; ^&^*P* < 0.05 NS vs. HS; ^*^P < 0.05 NS vs. NS-TG; ^#^*p* < 0.05 NS vs. HS (1-way ANOVA Newman-Keuls multiple-comparison test).

Similar to previous reports, PVN-RVLM neurons lacked spontaneous discharge at resting *V*_*m*_ (Chen and Toney, 2009; Chen et al., 2010); however, depolarizing current injections consistently evoked repetitive action potential firing. The role of the ER Ca^2+^ store in regulating excitability of PVN-RVLM neurons was examined by comparing the relationship between graded current injections and the evoked discharge in the absence and presence of TG (0.5 μM). Figure 4A shows representative discharge responses to a 200 pA depolarizing current pulse in the absence (top) and presence of the ER Ca^2+^ ATPase inhibitor, TG (bottom). Under control conditions, firing frequency in response to a 200 pA current injection in neurons from NS rats (*n* = 8, 22 ± 2 Hz) was significantly lower than HS (*n* = 7, 34 ± 5 Hz, *P* < 0.05 vs. NS control) (Figures 4A,B, left vs. right). Interestingly, inhibition of the ER Ca^2+^ store via bath application of TG significantly increased firing frequency in the NS group (*n* = 6, 30 ± 4 Hz, *P* < 0.05 vs. NS control), but not HS (*n* = 6, 32 ± 6 Hz, *P* > 0.05 vs. HS control) (Figures 4A,B). Furthermore, the slope of firing frequency in response to graded current injection was significantly greater in HS neurons (0.16 ± 0.01, *P* < 0.05 vs. NS control) compared to NS group (0.10 ± 0.01) (Figures 4B,C). Bath application of TG significantly increased the slope of firing frequency in response to graded current injections in NS group (0.14 ± 0.01, *P* < 0.05 vs. NS control), yet had no significant effects on HS neurons (0.15 ± 0.01, *P* > 0.05 vs. HS control) (Figures 4B,C).

### HS diet reduces spike-frequency adaptation in PVN-RVLM neurons

**Figure 5:**
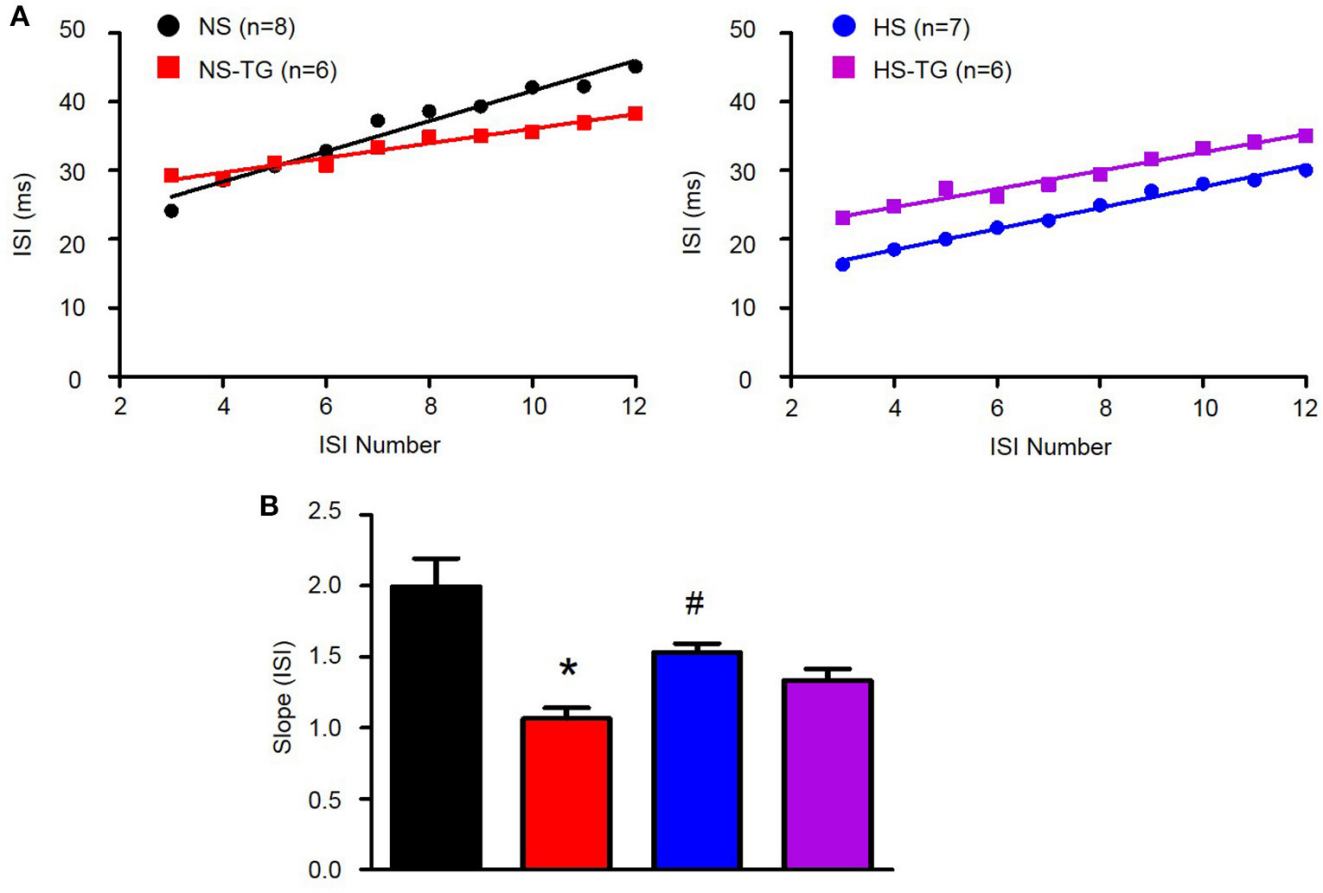
**Effects of ER Ca^**2+**^ uptake inhibition with TG on spike-frequency adaptation in PVN-RVLM neurons. (A)** Linear response of inter-spike interval (ISI)-ISI number over trains of action potentials in response to 200 pA current injection in PVN-RVLM neurons from NS left and HS right treatment groups before and after bath application of thapsigargin (TG). **(B)** Summary data showing slope of the ISI-ISI number response to 200 pA current injection was diminished in HS rats compared to NS revealing greater spike frequency adaptation in NS neurons. TG attenuated the slope of ISI in NS, but not HS neurons. NS-normal salt; HS-high salt; TG-thapsigargin. ^*^P < 0.05 NS vs. NS-TG; #*p* < 0.05 NS vs. HS (1-way ANOVA Newman-Keuls multiple-comparison test).

We have previously demonstrated that loss of SK channel function in AngII-salt HTN augments excitability of PVN-RVLM neurons through loss of spike-frequency adaptation (SFA) (Chen et al., 2010). Therefore, we examined whether the increased excitability of PVN-RVLM neurons in HS diet treated rats was associated with a decrease in SFA. Figure 5A illustrates the slope of the inter-spike interval (ISI)-ISI number curve over trains of action potentials in response to a 200 pA current injection in PVN-RVLM neurons from NS (left) and HS (right) rats. The slope of the linear fit of the ISI-ISI number curve was significantly (*P* < 0.05) greater in NS (2.2 ± 0.2, *P* < 0.05 vs. HS) compared to HS (1.5 ± 0.1) indicating that spike-frequency adaptation is reduced in HS neurons under control conditions (Figure 5B). Additionally, TG significantly decreased the slope of ISI-ISI number curve in NS (1.1 ± 0.1, *P* < 0.05 vs. NS control) group indicating that inhibition of the ER Ca^2+^ store reduces spike-frequency adaptation (Figure 5B). Interestingly, slope was unchanged in the HS group (1.3 ± 0.1, *P* > 0.05 vs. control) following application of TG (Figure 5B). Bath application of TG (0.5 μM) had no significant effects on any passive membrane properties (Table 2).

**Table 2:** **Passive membrane properties of PVN-RVLM neurons**.

**Group**	***n***	***V_*m*_* (mV)**	***C_m_* (pF)**	***R*_input_ (GΩ)**	***V_*t*_* (mV)**
NS	8	−59 ± 2	53 ± 4	0.62 ± 0.03	−42 ± 2
NS-TG	6	−59 ± 3	40 ± 5	0.78 ± 0.14	−44 ± 1
HS	7	−52 ± 2	40 ± 5	0.76 ± 0.08	−40 ± 1
HS-TG	6	−58 ± 3	48 ± 7	0.70 ± 0.10	−37 ± 2

*Values are mean ± SE, n, number of neurons; V_m_, resting membrane potential; C_m_, membrane capacitance; R_input_, depolarizing input resistance; V_t_, sub-threshold of membrane potential to fire action potential*.

### Histology

**Figure 6:**
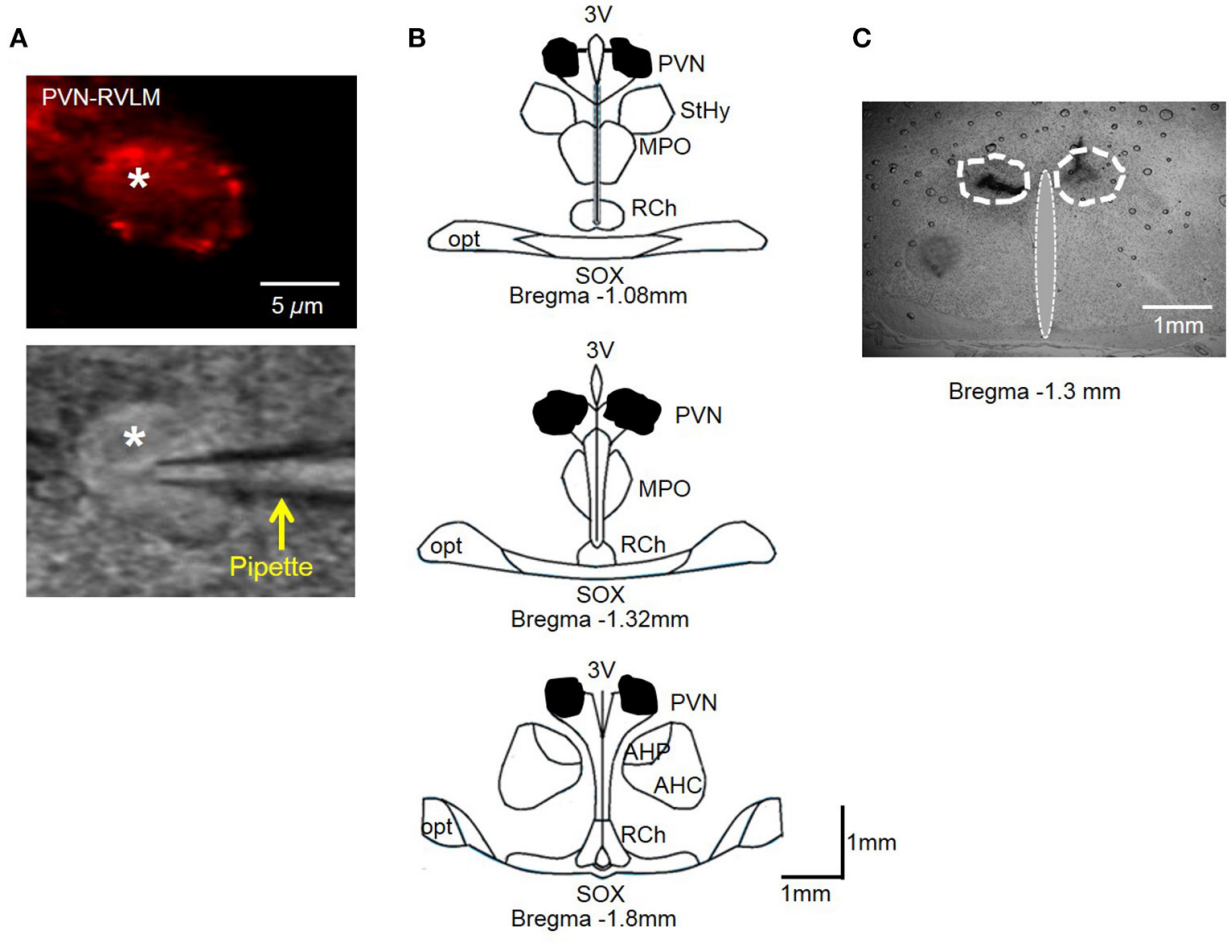
**(A)** PVN-RVLM neuron with red fluorescence from retrograde label (top) and same neuron with patch pipette positioned on cell surface with DIC microscopy (bottom). **(B)** Schematic drawings of coronal sections throughout the rat hypothalamus. Shaded area indicates spread of injected dye used to mark the injection sites in the bilateral PVN. The shape of each area was determined by overlaying tracings of the outermost diffusion area of injected dye (100 nl) through the rostral-caudal plane of the PVN. **(C)** Representative coronal slice through the PVN demonstrating spread of injected dye. AH, anterior hypothalamic area; 3V, third cerebral ventricle; RCh, retrochiasmatic area; MPO, medial preoptic nucleus; opt, optic tract; SOX, supraopticdecussation; StHy, striohypothalamic nucleus.

Injection sites were marked with 2% Chicago blue dye (100 nl). Coronal slices through the rostral caudal plane encompassing the PVN were examined to ensure microinjections were confined to the PVN as previously described by our laboratory (Larson et al., 2015a). Figure 6B shows the composite diffusion area of injected dye compiled by overlying brain slices from separate rats to demonstrate the outermost diffusion area. Distribution of injected dye was largely confined to the area encompassing the PVN (Figure 6C).

## Discussion

The PVN is a key regulatory center for SNA, and disinhibition of PVN neurons contributes to the augmented sympathetic outflow in neurogenic HTN. Here, we demonstrate the importance of PVN ER Ca^2+^ store function in regulating SNA and ABP *in vivo*, and neuronal excitability *in vitro*. Bilateral PVN microinjection of TG, the ER Ca^2+^ATPase inhibitor known to deplete the ER Ca^2+^ store (Thastrup et al., 1990), increased SSNA, RSNA, and MAP in a dose-dependent manner demonstrating that the ER Ca^2+^ store plays a significant role in modulating SNA and ABP. Interestingly, sympathoexcitatory responses to PVN TG were significantly blunted in rats fed a 2% HS diet for 5 weeks. Importantly, we also demonstrate increased neuronal excitability in PVN neurons with axon projections to the RVLM in rats fed a HS diet. Bath application of TG significantly increased excitability of PVN-RVLM neurons in rats with a NS diet yet had no significant effect in animals with a HS diet. These results indicate that inhibition of the ER Ca^2+^-ATPase and depletion of Ca^2+^ store likely plays a role in increasing PVN neuronal excitability, which may underlie the mechanisms of sympathoexcitation in rats with chronic HS intake.

Excess dietary salt is strongly associated with the incidence of cardiovascular diseases and is an important contributor to the pathogenesis of hypertension (Kotchen et al., 2013). We demonstrate that HS diet alone increases excitability of PVN-RVLM neurons in normotensive rats. This finding is consistent with recent evidence indicating that HS *per se* can increase the excitability of central autonomic networks (Stocker et al., 2010). Several studies indicate that sympathetic and pressor responses to excitatory and inhibitory stimulation of the RVLM are exaggerated by HS intake (Pawloski-Dahm and Gordon, 1993; Adams et al., 2007, 2008). Further evidence demonstrates that the enhanced excitability of central autonomic circuits contributes to exaggerated SNA and ABP responses during activation of physiological reflexes including the exercise pressor reflex (Yamauchi et al., 2014), stimulation of sciatic afferents (Pawloski-Dahm and Gordon, 1993; Simmonds et al., 2014), and ICV hypertonic saline (Simmonds et al., 2014) or AngII (Mann et al., 1980). Interestingly, depressor responses to aortic depressor nerve stimulation and acute volume expansion were also augmented by HS intake (Pawloski-Dahm and Gordon, 1993; Simmonds et al., 2014). Further studies have demonstrated the importance of the forebrain CVO's in mediating this response (Adams et al., 2009) and lesions of the anteroventral third ventricular (AV3V) region abolish the effects of salt on autonomic reflex activation (Simmonds et al., 2014). Collectively, these results indicated that HS intake sensitizes central autonomic circuitry even in the absence of hypertension, and we are the first to report that HS diet augments excitability of pre-sympathetic PVN neurons in normotensive rats. Enhanced neuronal activity among PVN neurons has been noted in a variety of cardiovascular diseases characterized by salt retention and high sympathetic outflow including heart failure and salt-sensitive HTN (Patel, 2000; Allen, 2002). Similar to previous findings (Stocker et al., 2010), we speculate that increases in PVN neuronal excitability due to HS intake may increase the impact of excitatory synaptic inputs to the PVN creating a predisposition for HTN.

Previous studies have demonstrated that cerebral spinal fluid (CSF) Na^+^ levels are not elevated in salt-resistant Sprague Dawley rats fed a HS diet (Nakamura and Cowley, 1989; Huang et al., 2004). These results suggest that mechanism other than direct Na^+^ stimulation on PVN neurons likely contributes to the increase in the *in vitro* excitability of pre-sympathetic PVN neurons and *in vivo* sympathoexcitation in normotensive rats with HS intake. Moreover, we have reported that sympathoexcitatory responses to intra-carotid artery (ICA) infusion of hypertonic saline are attenuated by PVN angiotensin II type 1 receptor blockade suggesting that synaptic activity in the PVN is required for HS induced sympathetic activation (Chen and Toney, 2001). Furthermore, central hyperosmotic stimulation increases c-fos expression in PVN-projecting OVLT neurons in a concentration dependent manner (Shi et al., 2008) and electric lesions of the OVLT significantly attenuated sympathoexcitatory responses to ICA infusion of hypertonic NaCl (Shi et al., 2007). One recent study reported that OVLT neurons expressed a concentration dependent increase in neuronal activity in response to hypertonic NaCl stimulation, and OVLT microinjection of hypertonic NaCl produced graded increases in SNA and ABP (Kinsman et al., 2017). Collectively, synaptic activity from Na^+^ or osmolality sensitive neurons in the OVLT likely plays a significant role in altering PVN neuronal excitability in response to HS intake.

Although some neuronal pathways have been identified to be involved in the sympathoexcitation in response to HS intake, less is known about the cellular and molecular mechanisms responsible for augmented PVN neuronal excitability due to HS intake. Our lab has recently demonstrated that dysfunction of SK channels in the pre-sympathetic PVN-RVLM neurons contributes to the augmented neuronal excitability and sympathoexcitation in AngII-salt HTN using both *in vitro* and *in vivo* approach (Chen et al., 2010; Larson et al., 2015a). Furthermore, we showed that SK channel dysfunction is present in normotensive rats fed a 2% HS diet (Larson et al., 2015a). Interestingly, depletion of ER Ca^2+^ store via bilateral microinjection of TG, an inhibitor of the ER Ca^2+^ ATPase, significantly increased SSNA, RSNA, and MAP. It's important to note that this response demonstrated dose dependence helping to rule out non-specific drug actions that potentially contribute to sympathoexcitation. Additionally, we performed control experiments by delivering TG via IV catheter, and microinjection outside the PVN (2.5 mm lateral to midline) in separate experiments. Neither of these two control deliveries resulted in a significant change in SNA or ABP, which further demonstrates that action of the drug in our acute experimental model likely depends on actions within the PVN. Furthermore, spread of injected dye (100 nl) following experiments was largely confined to the PVN.

In order to further investigate the underlying mechanisms of ER Ca^2+^ stores in the PVN in regulating SNA and ABP, we utilized whole-cell current clamp to examine excitability of pre-sympathetic PVN-RVLM neurons identified by retrograde labeling under brain slice preparation. Inhibition of the ER Ca^2+^ store via bath application of TG increased excitability of PVN-RVLM neurons (Figure 4). TG is an inhibitor of the ER Ca^2+^ ATPase which pumps Ca^2+^ into the ER to maintain high Ca^2+^ levels within the ER and preserves intra-neuronal Ca^2+^ homeostasis (Thastrup et al., 1990). There are several possible mechanisms whereby inhibition of the ER Ca^2+^ ATPase could influence neuronal excitability. Initially, TG causes an increase in intracellular Ca^2+^ due to the inability of the ER to remove excess cytosolic Ca^2+^ as well as activation of store operated Ca^2+^ channels (Putney, 2003; Brini et al., 2014). Secondly, depletion of the ER Ca^2+^ store by TG could contribute to the dysfunction of Ca^2+^ activated K^+^ channels including SK channels. Available evidence supports the latter possibility. First, a previous study in sympathetic neurons showed that despite an initial rise in intracellular Ca^2+^, action potential evoked Ca^2+^ transients were significantly diminished with TG treatment (Akita and Kuba, 2000). The diminished Ca^2+^ transient significantly reduced the SK channel mediated after-hyperpolarization potential with a peak response revealed 30 min following TG treatment. Our results display a similar time course with maximum sympathexcitatory response occurring 35–40 min after microinjection of TG. Secondly, TG inhibits spike frequency adaptation (SFA) among PVN-RVLM neurons contributing to increased excitability (Figure 5), and we have previously reported that reductions in SFA contribute to the increased excitability of PVN-RVLM neurons following SK channel blockade (Chen and Toney, 2009). Thirdly, we have also previously demonstrated that Ca^2+^ free bath application significantly diminished SK currents and blockade of SK channels increased excitability among PVN-RVLM neurons (Chen and Toney, 2009). Finally, our recent findings showing that Ca^2+^ chelators BAPTA and EGTA, significantly increased both *in vivo* SNA and *in vitro* neuronal excitability of PVN-RVLM neurons also support this possibility (Larson et al., 2016). Whether reduced SK currents underlie the mechanisms of TG elicited increases in excitability of PVN-RVLM neurons remains to be studied in the future.

The ER is an important regulator of intracellular Ca^2+^ homeostasis. Here, we also present a novel mechanism whereby HS diet disrupts ER Ca^2+^ stores in the pre-sympathetic PVN neurons. SSNA, RSNA, and MAP responses to PVN microinjection of TG were significantly attenuated in HS rats indicating that Ca^2+^ store function may be altered by HS treatment. Interesting, we also report that firing frequency in response to a 200 pA current injection is significantly increased in PVN-RVLM neurons in the HS treatment group. Additionally, the slope of the linear response to graded current injections is significantly augmented in neurons from the HS treatment group further indicating an increase in excitability (Figure 4). We have previously reported that excitability of PVN-RVLM neurons is augmented in AngII-salt HTN (Chen et al., 2010), and results from the present study indicate the HS diet likely plays a critical role in augmenting PVN neuronal excitability. Interestingly, bath application of TG had no significant effect on either firing frequency in response to 200 pA current injection, or the slope of firing frequency in response to graded current injection in HS rats (Figure 4). These data implicate a possible role for altered ER Ca^2+^ store function that likely contributes to augmented PVN neuronal excitability in HS rats. In order to explore possible mechanisms for the augmented excitability of PVN-RVLM neurons due to HS intake, we compared SFA in NS and HS neurons. SFA was significantly attenuated in HS neurons indicated by a reduction in the slope of ISI-ISI number curve and bath application of TG did not significantly alter SFA in HS neurons. SK channel is Ca^2+^ dependent and a primary mediator of SFA (Sah, 1996; Stocker et al., 1999). Therefore, we expect that the reduced SFA in HS neurons could be due to reduced SK channel activation through diminished ER Ca^2+^ signaling.

We utilized anesthetized whole animal preparations to examine the effects of PVN microinjection of TG in rats. It should be recognized that the use of anesthesia is a limitation due to previously established differences in sympathetic responses between anesthetized and conscious rats (Kannan et al., 1987, 1989). We used a microinjection volume of 100 nL and it's possible that actions of TG outside of the PVN contributed to the response. Our data indicate that spread of the same volume of injected dye was confined to the PVN region and control injections failed to elicit a response.

In summary, we demonstrate that inhibition of PVN ER Ca^2+^-ATPase and depletion of Ca^2+^ store from the ER with TG significantly augments SNA and MAP *in vivo*, and excitability of PVN-RVLM neurons *in vitro*. Additionally, we report that HS diet increases excitability of PVN-RVLM neurons. Interestingly, sympathoexcitatory responses to PVN microinjection of TG were attenuated and bath application of TG did not significantly increase neuronal excitability in rats fed a HS diet indicating that altered ER Ca^2+^ handling likely plays a role in the augmented neuronal excitability. Collectively, the present study suggest that HS diet *per se* augments the excitability of pre-sympathetic PVN neurons, which may underlie the mechanisms of sympathoexcitation due to HS intake.

## Perspectives

Excess dietary salt intake is a major risk factor for the development of cardiovascular disease even in the absence of over HTN. Recent studies indicate that HS intake sensitizes central neural circuitry contributing to exaggerated SNA and pressor responses to physiological stimuli. The present study identifies a novel mechanism whereby disruption of Ca^2+^ homeostasis in the ER likely plays a role in augmented pre-sympathetic PVN neuronal excitability *in vitro* and sympathoexcitation *in vivo* due to HS diet. The ER may be a new and novel treatment target due to its involvement in a multitude of cellular process and we are only beginning to understand the role it plays in mediating PVN neuronal excitability and sympathetic activation. Future studies are needed to elucidate the molecular signaling mechanisms that underlie HS diet induced ER dysfunction.

## Author contributions

RL, LG, and MH performed *in vivo* microinjection experiments; AC performed *in vitro* whole cell patch clamp recordings. RL, AC, MH, LG, and QC analyzed data; RL, AC, LG, and QC prepared figures. RL and AC drafted manuscript; RL and QC edited and revised manuscript; RL, AC, MH, LG, ZC, ZS, and QC approved the final version of manuscript; RL, AC, ZC, ZS, and QC conceptualized and designed the research.

## Funding

This study was funded by The American Heart Association 11SDG7420029 (ZS) 10SDG2640130 (QC) and The National Institute of Health HL122952 (QC).

### Conflict of interest statement

The authors declare that the research was conducted in the absence of any commercial or financial relationships that could be construed as a potential conflict of interest. The reviewer HZ and handling Editor declared their shared affiliation, and the handling Editor states that the process nevertheless met the standards of a fair and objective review.
